# Binge Drinking Effects on EEG in Young Adult Humans

**DOI:** 10.3390/ijerph7052325

**Published:** 2010-05-10

**Authors:** Kelly E. Courtney, John Polich

**Affiliations:** The Scripps Research Institute, Molecular and Integrative Neurosciences Department, La Jolla, CA 92037, USA; E-Mail: kellyc@scripps.edu

**Keywords:** binge drinking, CNS, EEG, young adults, undergraduates

## Abstract

Young adult (N = 96) university students who varied in their binge drinking history were assessed by electroencephalography (EEG) recording during passive viewing. Groups consisted of male and female non-binge drinkers (>1 to 5/4 drinks/ounces in under two hours), low-binge drinkers (5/4–7/6 drinks/ounces in under two hours), and high-binge drinkers (≥ 10 drinks/ounces in under two hours), who had been drinking alcohol at their respective levels for an average of 3 years. The non- and low-binge drinkers exhibited less spectral power than the high-binge drinkers in the delta (0–4 Hz) and fast-beta (20–35 Hz) bands. Binge drinking appears to be associated with a specific pattern of brain electrical activity in young adults that may reflect the future development of alcoholism.

## 1. Introduction

Binge drinking is a social phenomenon with a high prevalence among undergraduate students. The College Alcohol Study (CAS) found that for a sample of 140 colleges in the United States, 44% of the responding students were binge (≥5/4 successive drinks/ounces for males and females, respectively) drinkers [1]. The neurocognitive effects of this pattern of alcohol intake have not been well studied, but the varied literature suggests several negative sequelae [2]. Although an empirical definition of binge drinking has not been used consistently, cognitive and behavioral studies generally have found frontal lobe and working memory deficits. Heavy social drinkers, defined to include those who engaged in binge drinking episodes, demonstrated delayed auditory and verbal memory deficits related to task difficulty that were absent for light social drinkers [3]. However, the discrepancies between social and binge patterns of alcohol consumption imply that these results should be cautiously extrapolated to the binge drinking population. Other neurocognitive impairments such as deficits in the Paced Auditory Serial Addition Test, executive planning function, episodic memory [4], spatial working memory, and pattern recognition task impairments also have been associated with binge drinking [5]. Thus, the binge drinking pattern of alcohol consumption may place individuals at an increased risk for acquiring alcohol-related cognitive impairments [3].

Assessment of binge drinking in young adults using electroencephalography (EEG) has not been reported, but findings from cognitive event-related potential (ERP) studies are suggestive. Maurage *et al.* [6] employed an auditory stimulus valence detection paradigm in which first-year undergraduates were instructed to discriminate between negative and positive auditory stimuli (e.g., the semantically neutral word “paper” was read by a male or female voice with angry or happy prosody). ERPs were collected at the beginning and end of the academic year. Peak latency of P1, N2, and P3b components were more delayed in latency as binge drinking increased over the year. However, the binge-drinking cutoff employed was quite high at 200 grams pure ethanol/week (≈24 ounces of hard alcohol). The typical young adult binge drinker does not consume alcohol at regular weekly intervals, and this irregularity of ethanol consumption is a major characteristic of the binge consumption and withdrawal pattern [2]. Ehlers *et al.* [7] utilized a facial discrimination task in which digital photographs of happy, neutral, and sad faces were presented, with participants instructed to indicate with a button press when the happy and sad stimuli appeared while ignoring the neutral stimuli. Adolescents exposed to alcohol (≥5 drinks/occasion ≈ 5 ounces) produced smaller P300 amplitudes, with a positive family history for alcohol dependence found to be a significant covariate. P300 latency also was decreased for alcohol and drug-exposed young adults relative to controls. These findings imply that binge drinking and related variables can affect neuroelectric measures related to cognitive processing.

### 1.1. EEG and Alcoholism

The majority of EEG investigations of alcohol drinkers have focused on alcoholics or individuals with a family history of alcoholism. These results also are difficult to extend to binge drinking populations as the related variables confound alcohol consumption effects for EEG measures. However, EEG studies of alcoholics have demonstrated decreased alpha band (8–12 Hz) power [8,9], increased beta band (12–30 Hz) power [10,11], and the presence of low-voltage fast (<20 μV, 14–30 Hz) EEG patterns [12,13]. These low-voltage fast desynchronized patterns have been interpreted as reflecting hyperarousal of the central nervous system (CNS) [10]. Hyperarousal of the CNS in alcoholics has been substantiated by the discovery of a corresponding elevation in cardiac output, which suggests that these individuals require greater sedation to achieve a “normal” arousal level [14]. Increasing the amount of alcohol consumption is associated with greater amounts of alpha activity and slowing of the predominant alpha frequency [15,16]. CNS hyperarousal is indexed by high-frequency beta (19.5–39.8 Hz) activity, which has been used to predict relapse rates among abstinent alcoholics [14,17] and differentiate between abstinent and non-abstinent alcoholics [10]. Taken together, binge drinking and chronic alcoholism may represent two stages of the same phenomenon [18], such that similar CNS hyperarousal may be a biomarker for the binge drinking population.

Alcoholism is a highly heritable disorder with heritability estimates of susceptibility between 50% and 60% [19]. Alcohol expectancies—*i.e.*, beliefs about the drug’s impact on behavior—and subjective experience have been shown to be a genetically influenced characteristic having a heritability factor between 0.4 and 0.6 [20,21], with greater alcohol consumption found for high-risk compared to low-risk families [22]. The distinctive resting EEG pattern of an individual tends to be highly heritable and stable [23,24], with the average heritability for delta, theta, alpha, and beta frequencies at 76%, 89%, 89%, and 86%, respectively [25]. Hence, an “alcoholism phenotype” may be observed in the EEG of high-risk children, although EEG power spectra findings from low- and high-risk for alcoholism descendants are variable: Children of alcoholic parents have more fast (beta) activity than children without alcoholic parents [26], while no differences in baseline EEG variables in the high frequency range were found for older (19–25) populations [27,28]. Thus, familial alcoholism covaries with behavioral and neuroimaging measures of binge drinking [7,29] and is an important background variable to consider when investigating binge drinking’s CNS effects.

### 1.2. EEG and Drinking Amount

The effects of chronic alcohol intake amount on subsequent normal CNS neuroelectric activity have been little studied, although there are intriguing hints. High-alcohol drinking (HAD) rats demonstrated greater spectral power for the delta (2–6 Hz) and theta (4–6 Hz) bands compared to low-alcohol drinking (LAD) rats, with both groups specifically bred for their drinking proclivity. Further, HAD rats exhibited increased activity in the fast beta (13–30 Hz) and high-voltage fast alpha (9–12 Hz) bands [30]. These findings are consistent with EEG effects observed in binge drinkers who had not consumed alcohol at the frequency that is normally associated with alcoholism.

There are few studies using “normal” adult human drinkers who vary in their drinking levels (for a review, see Courtney & Polich, 2009). Ehlers and colleagues [28,31] assessed EEG in family history negative “low” and “moderate” drinkers (with drinking level determined by the quantity of alcohol consumption times the frequency of alcohol consumption). Moderate drinkers (scoring ≥ 40) evinced greater mean spectral power and higher peak frequency in the beta (12–20 Hz) band at baseline, so that beta activity may index quantity and frequency of alcohol consumption. Bruin *et al.* [32] quantified EEG synchronization in heavy drinking college students, defined as those who consume more than 30 units containing 12 gram of alcohol per week (≈ 30 ounces of hard alcohol). Functional connectivity during eyes closed recording differed between light and heavy drinkers, such that heavy drinkers exhibited abnormally increased synchronization in the theta (4–8 Hz) and gamma (30–45 Hz) bands as indexed by significant synchronization likelihood comparisons across groups. Both bands have been associated with memory formation as subserved by hippocampo-neocortical connections [33], and the synchronization of the heavy drinkers in these bands could reflect changes in the hippocampus, cortex, and/or hippocampo-neocortical connections as observed for alcoholics [34,35]. Thus, several lines of evidence converge on the possibility that “normal” alcohol consumption can alter EEG signals.

### 1.3. Present Study

The major goal of the present study was to assess resting EEG in young adults who varied in their alcohol-drinking amount in the absence of a family history for alcoholism. Binge drinking was defined as a pattern of alcohol drinking that brings the blood alcohol concentration (BAC) to 0.08 gram percent or above more than once within the past 6 months. Achieving this level requires 5 or more drinks (≥5 ounces) for males and 4 or more drinks (≥ 4 ounces) for females are consumed within a 2 hour period [2]. To assay possible EEG changes from past alcohol consumption, 3 binge-drinking groups with equal numbers of each gender were obtained: non-binging (>1 to <5/4 alcoholic drinks/ounces within 2 hours and occurring within the past 6 months), low-binging (5/4–7/6 drinks/ounces within 2 hours on more than 1 occasion within the past 6 months), and high-binging (≥10 drinks/ounces within 2 hours on more than 1 occasion within the past 6 months) alcohol drinkers. This approach will assess binge-drinking correlated CNS neuroelectric activity in young adults of both genders unrelated to alcoholics.

## 2. Results and Discussion

### 2.1. Demographics

Table 1 summarizes the demographic variables for each binge and sex group. Separate 3 binge × 2 sex group analyses of variance were conducted on each factor. Age [*F*(1, 90) = 8.0, *p* < 0.05], height [*F*(1, 90) = 18.1, *p* < 0.001], and weight [*F*(1, 90) = 18.2, *p* < 0.001] differed between sex but did not differ among binge groups (*p*s > 0.05). The groups did not differ on the total number of years consuming alcohol, although non-binging females had not been drinking as long as non-binging males which produced a reliable interaction [*F*(2, 90) = 3.6, (*p* = 0.03)]. As expected, the drinking variables varied among groups, with the high-binge drinkers consuming more alcohol on more days per month, more alcohol per time, more alcohol in two hours, and a higher binge frequency than non-binge drinkers (*p*s < 0.001). High-binge drinkers also drank more alcohol per occasion, more alcohol in two hours, and had a higher binge frequency than low-binge drinkers (*p*s < 0.05). The low-binge drinkers consumed more alcohol on more days per month, more alcohol per time, more alcohol in two hours, and a higher binge frequency than the non-binge drinkers (*p*s < 0.05). The drink days per month variable also varied between the sexes, with the males drinking alcohol on more days per month than the females [*F*(1, 90) = 4.6, *p* = 0.03].

### 2.2. Spectral Power Findings

**Figure 1.**
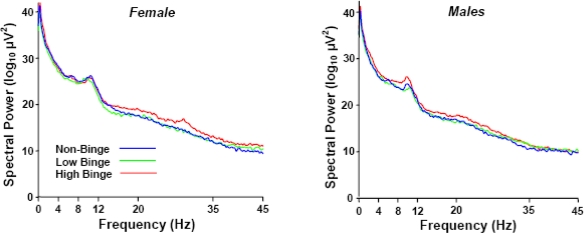
Grand average power spectra of each binge and sex group as a function of EEG.

Figure 1 illustrates the grand-averaged power spectra (Cz electrode) of each binge and sex group as a function of brainwave frequency. The abscissa points define the EEG bands assayed. Note the high-binge subjects demonstrate greater spectral power in the lower delta (0–4 Hz) and upper beta (20–35 Hz) frequency bands. The increase in power in the alpha (8–12 Hz) band is typical for both female and male subjects but did not differ between the sexes or among groups.

**Figure 2.**
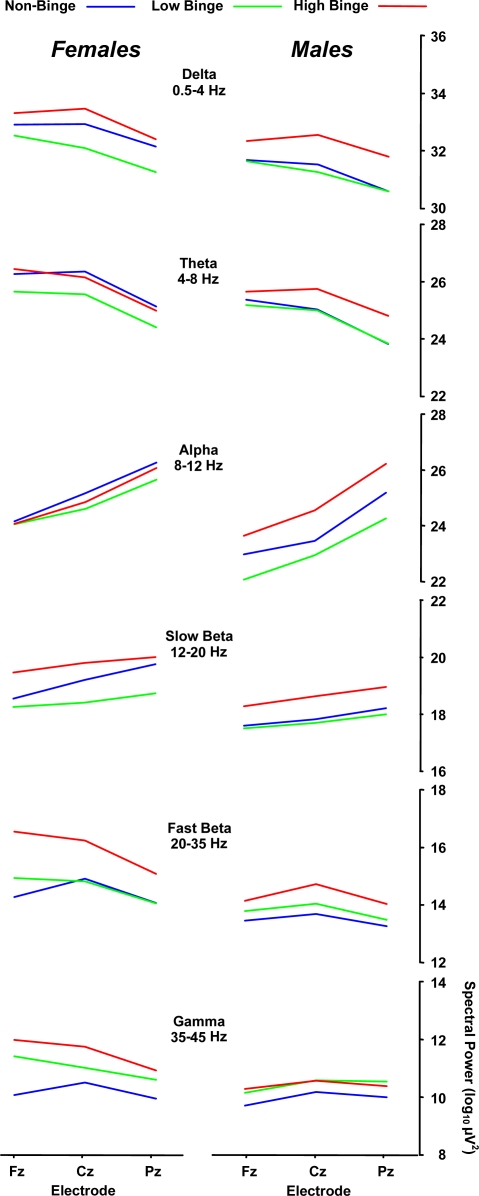
Mean spectral power for each binge group and subject sex as a functjion of midline electrode for each EEC frequency band.

Table 2 summarizes the results from a 3 binge × 2 sex × 3 midline electrode analysis of variance applied to the spectral power data from each EEG band. Partial *η*^2^ is the proportion of non-error variance accounted for by each variable separately. Post-hoc mean comparisons were conducted with the Scheffé procedure. Figure 2 illustrates the mean spectral power of each binge and sex group as a function of midline electrode location (Fz, Cz, Pz) for each EEG band.

### 2.3. Delta (0–4 Hz) Band

Delta band power was significantly different among the binge and between the sex groups. Post-hoc assessment revealed that the high-binge group exhibited greater spectral mean power compared to the low-binge group (*p* = 0.008), with high-binge drinkers having greater power than low-binge drinkers across electrodes (32.6 *vs.* 31.6 μV^2^). No reliable effects for the non-binge relative to binge groups were obtained (*p* > 0.10 in all cases). Separate post-hoc analyses produced the same statistical pattern for each sex group. It is noteworthy that (the non-significant) greater delta power for females compared to males appear to have contributed to the outcome patterns by increasing the group differences for the high-binge subjects more for the females than the males. These differences may reflect the different alcohol consumption levels used to define binge drinking for each sex [2].

### 2.4. Beta (12–35 Hz)

The fast-beta spectral power yielded a significant binge group main effect, and post-hoc assessment found that the high-binge group exhibited greater mean spectral power than the non-binge group (15.1 *vs.* 13.9 μV^2^, *p* = 0.03), with a marginal difference obtained between high- and low-binge drinkers (15.1 *vs.* 14.2 μV^2^, *p* = 0.10). These findings are consistent with those from previous studies reporting that alcoholics exhibit increased EEG spectral power in the beta band [10,11,36]. High-binge drinkers who are not alcohol-dependent may therefore exhibit EEG power variation predictive of future alcohol dependency. In this context, it is reasonable to suggest that high-binge drinkers may possess an overactive CNS and therefore consume greater quantities of alcohol at any one sitting to suppress their CNS activity [14]. Even though the participants of the present study were not alcohol dependent and free from alcohol dependency in their family, the fast-beta band results suggest that they may be at-risk for future alcohol dependency.

### 2.5. Sex Effects

The delta, theta, slow-beta, and fast-beta bands yielded reliable differences for sex: females exhibited greater spectral power compared to males (≈1 μV^2^). The interactions between sex and electrode for the fast beta and gamma bands are due to the decrease in power from the frontal to parietal electrodes for the female compared to the lack of change for the male subjects. The origins of these effects are unclear but may be related to a greater sensitivity to high levels of alcohol consumption for female compared to male young adults.

## 3. Experimental Section

### 3.1. Participants

Participants were recruited from the University of California, San Diego (see Table 1), with individuals identified by responses to a questionnaire distributed to undergraduate psychology classes. Survey completion was optional, and the information obtained was used to characterize alcohol-drinking patterns. A follow-up structured telephone interview excluded individuals reporting personal/familial (up to two generations) alcoholism, neurologic/psychiatric disorders, and recent recreational drug use. Exclusionary criteria also included not consuming alcohol, tobacco smoking, use of psychiatric medication, and serious health problems (e.g., asthma, heart condition, *etc*.). A third survey after written consent ensured current medical health and capability at the time of testing.

Binge-groups consisted of male/female alcohol drinkers that were non-binging (>1 but <5/4 alcoholic drinks/ounces within 2 hours and occurring within the past 6 months), low-binging (5/4–7/6 drinks/ounces within 2 hours on more than 1 occasion within the past 6 months), and high-binging (≥10 drinks/ounces within 2 hours on more than 1 occasion within the past 6 months). Equal numbers of each sex and n = 16 in each binge group were obtained (total N = 96). Participants were matched on educational level, handedness, and demographic background. Participants were instructed to refrain from any alcohol consumption and drug use for at least 48 hours prior to testing.

### 3.2. Recording Conditions

EEG recordings were obtained from 21 channels at the Fz, FCz, Cz, CPz, and Pz central recording sites, the Fp1/Fp2, F3/F4, C3/C4, P3/P4, O1/O2 medial hemispheric sites, as well as F7/F8, T7/T8, P7/P8 lateral hemispheric sites of the modified 10–20 system [37]. An Electro-cap system was used, with additional tin electrodes affixed with paste and tape. Cephalic electrodes were referenced to linked mastoids balanced for resistance, with a forehead ground. These methods permit a direct comparison to previous studies and do not compromise hemispheric difference measures. Impedance for all recording sites was 10 kΩ or less. Electro-ocular (EOG) activity was monitored with electrodes placed at the outer canthus and infra-orbitally about the left eye. EEG was recorded for 3 minutes with eyes open as is typically done to avoid a preponderance of alpha activity, and the subject sitting in a sound-attenuated booth. The bandpass was 0.02–45 Hz, with a digitization rate of 256 Hz employed. Trials where the amplitude exceeded 100 μV were excluded from analysis.

### 3.3. Analysis Procedures

A total of 80 seconds of artifact-free EEG data (< ±100 μV) were selected from each recording and spectral analysis was used to extract mean spectral power and mean frequency (defined by obtaining the frequency of each .25 Hz segment within a band and dividing by the number of segments) in six bands: delta (0–4 Hz), theta (4–8 Hz), alpha (8–12 Hz), slow-beta (12–20 Hz), fast-beta (20–35 Hz), and gamma (35–45 Hz). The spectral power data (μV^2^) were subjected to a log_10_ transformation [38], with analysis of variance applied using a mixed model repeated measures design. Between-subject factors were binge group membership with 3 levels (non-, low-, high-binging) and the 2 sexes (male, female). Midline electrodes was a repeated measures factor with 3 levels (Fz, Cz, Pz). Greenhouse-Geisser corrections were applied to the repeated measures factor with 3 or more levels, with the uncorrected *df* reported. Homogeneity of variance tests were conducted for the between-subjects factors.

Preliminary analyses on data from the lateral electrodes found no major effects of interest different from the midline recording sites. In addition, all analyses were conducted twice to examine possible covariate effects of binge drinking *frequency*. This factor did not contribute to binge group spectral power and is not considered further. Mean spectral band frequency also was assessed, produced no significant results, and is not considered further.

## 4. Conclusions

The present study was designed to characterize how binge drinking affects CNS neuroelectric activity in male and female undergraduate participants who were carefully screened for the presence of covariates. EEG recorded in a passive, eyes-open procedure indicated enhanced spectral power in the delta (0–4 Hz) and fast-beta (20–35 Hz) bands for the high-binge drinkers. Although the causal relationship between binge drinking and increased fast-beta power remains unclear, the alteration of fast-beta activity suggests that high-binge drinkers exhibit a similar EEG spectral pattern as alcoholics. Thus, relative increases in fast-beta power may be a biomarker for potential future alcoholism even in the absence of familial alcoholism.

The motivation for this investigation stemmed from a need for empirical evaluation of binge drinking. Although experimental “binge” drinking studies have been conducted, none have examined factors known to correlate with binge drinking and few employ a reliable definition of this unique alcohol consumption pattern [2]. The present approach assessed these issues in an attempt to delineate how binge-drinking EEG patterns might differentially index CNS processing related to the development of alcoholism.

### 4.1. Limitations and Future Directions

Although the present study was designed to examine binge drinking in young adults, the source of the participant sample may be a limitation as all subjects were obtained from the University of California, San Diego—a multicultural institution. To address this issue, all alcohol abstainers were excluded, all individuals who reported a “flushing” response from alcohol were excluded, and all individuals with any family history for alcoholism were excluded. Hence, considerable perspective was applied toward the definition and selection of the samples employed. In addition, self-report measures are generally accepted as reliable [39–41], but it has been argued that self-report binge drinking quantifications may not reflect actual amounts of consumption [42]. Repeated assessments were therefore obtained after strong assurances of confidentiality were proffered, with clear and direct self-report questions stated to facilitate alcohol consumption measure accuracy.

The ultimate goal of this study is to enhance the knowledge base of alcohol research. The lack of information on the psychophysiological consequences of binge drinking may be one factor contributing to its increasing incidence and prevalence rates. The present findings suggest that binge drinking is not just harmless social fun, but if sustained may lead to alcohol dependency later in life.

## Figures and Tables

**Table 1.** Summary of demographic factors for each binge group (mean [SD]).

	Non-Binge (>1 to 5/4 drinks within 2 hrs)		Low-Binge (5/4 to 7/6 drinks within 2 hrs)		High-Binge (≥10 drinks within 2 hrs)	
	Female	Male	Female	Male	Female	Male

Sample Size (n)	16	16	16	16	16	16
Age (years)	20.38 [1.26]	21.81 [1.68]	20.38 [1.15]	20.50 [0.97]	19.94 [1.12]	20.81 [1.97]
Education (college)	2.93 [1.07]	3.44 [0.81]	3.14 [1.03]	3.29 [0.83]	2.77 [0.93]	3 [1.36]
Height (ft)	5.38 [0.23]	5.75 [0.32]	5.45 [0.44]	5.76 [0.37]	5.33 [0.39]	5.59 [0.40]
Weight (pounds)	127.88 [30.75]	163.44 [26.76]	148.93 [26.11]	159.47 [27.99]	143 [24.15]	166.75 [22.37]
Years Drinking	1.91 [0.97]	3.69 [2.55]	2.88 [1.50]	2.69 [1.59]	3.13 [1.54]	3.06 [1.24]
Drink Days/Month	3 [2.47]	4 [4.00]	4.81 [2.46]	8 [7.58]	7.69 [3.55]	10 [4.89]
Drinks/Occasion	3 [0.98]	3 [1.54]	5 [3.12]	5 [2.28]	6.75 [2.67]	6 [2.85]
Amount: Drinks/2 hrs, Past 6 Months	3 [1.07]	3 [1.35]	5.57 [0.85]	6 [1.26]	10 [3.61]	11 [3.76]
Binge Frequency: Past 6 months	0.11 [0.33]	0 [0]	9.43 [14.71]	7 [6.42]	14 [14.37]	23 [23.72]

**Table 2.** Summary of ANOVAs on the Mean Spectral Power from each EEG Band and Partial *η*^2^.

Factor (df)	Binge Group (2,90)		Sex (1,90)		Electrode (2,180)		BG × S (2,90)		BG × E (4,180)		S × E (2,180)		BG × S × E (4,180)	
	*F*	*η*^2^	*F*	*η*^2^	*F*	*η*^2^	*F*	*η*^2^	*F*	*η*^2^	*F*	*η*^2^	*F*	*η*^2^

Delta (0–4 Hz)	5.1**	0.10	13.0***	0.13	22.7***	0.20	---	---	---	---	---	---	---	---
Theta (4–8 Hz)	---	---	3.9*	0.04	135.8***	0.60	---	---	---	---	---	---	---	---
Alpha (8–12 Hz)	---	---	---	---	136.3***	0.60	---	---	---	---	---	---	---	---
Slow–Beta (12–20 Hz)	---	---	6.8*	0.07	7.5***	0.08	---	---	---	---	---	---	---	---
Fast–Beta (20–35 Hz)	3.9*	0.08	11.2**	0.11	20.5***	0.19	---	---	---	---	4.2*	0.05	---	---
Gamma (35–45 Hz)	---	---	---	---	3.5*	0.04	---	---	---	---	5.5**	0.06	---	---

* p *< 0.05,*

** p *< 0.01,*

*** p *< 0.001.*
